# The LUSBI Protocol (Lung Ultrasound/BREST Score/Inferior Vena Cava)—Its Role in a Differential Diagnostic Approach to Dyspnea of Cardiogenic and Non-Cardiogenic Origin

**DOI:** 10.3390/medicina60091521

**Published:** 2024-09-18

**Authors:** Boris Dojcinovic, Nada Banjac, Sasa Vukmirovic, Tamara Dojcinovic, Lucija V. Vasovic, Dalibor Mihajlovic, Velibor Vasovic

**Affiliations:** 1 Emergency Medical Service of Primary Health Care Center in Banja Luka, 78000 Banja Luka, Bosnia and Herzegovina; 2 Medical Faculty, University of Banja Luka, 78000 Banja Luka, Bosnia and Herzegovina; 3 Department of Pharmacology, Toxicology and Clinical Pharmacology, Medical Faculty, University of Novi Sad, 21000 Novi Sad, Serbia; sasa.vukmirovic@mf.uns.ac.rs (S.V.);; 4 Internal Medicine Clinic, University Clinical Center of the Republic of Srpska, 78000 Banja Luka, Bosnia and Herzegovina; 5 Institute for Pulmonary Diseases of Vojvodina, 21000 Novi Sad, Serbia; vasovicl720@gmail.com; 6 Medical Faculty, University of Novi Sad, 21000 Novi Sad, Serbia

**Keywords:** lung ultrasound, heart failure, LUSBI protocol, BREST score, inferior vena cava

## Abstract

*Background and Objectives*: PoCUS ultrasound applications are widely used in everyday work, especially in the field of emergency medicine. The main goal of this research was to create a diagnostic and therapeutic protocol that will integrate ultrasound examination of the lungs, ultrasound measurements of the inferior vena cava (assessment of central venous pressure) and BREST scores (risk stratification for heart failure), with the aim of establishing a more effective differential diagnostic approach for dyspneic patients. *Materials and Methods:* A cross-sectional study was conducted in the emergency medicine department with the educational center of the community health center of Banja Luka. Eighty patients of both sexes were included and divided into experimental and control groups based on the presence or absence of dyspnea as a dominant subjective complaint. Based on the abovementioned variables, the LUSBI protocol (lung ultrasound/BREST score/inferior vena cava) was created, including profiles to determine the nature of the origin of complaints. The biochemical marker of heart failure NT pro-BNP served as a laboratory confirmation of the cardiac origin of the complaints. *Results*: The distribution of NT pro BNP values in the experimental group showed statistically significant differences between individual profiles of the LUSBI protocol (*p* < 0.001). Patients assigned to group B PLAPS 2 had significantly higher average values of NT pro-BNP (20159.00 ± 3114.02 pg/mL) compared to other LUSBI profiles. Patients from the experimental group who had a high risk of heart failure according to their BREST scores also had a significantly higher average maximum expiratory diameter compared to those without heart failure (*p* = 0.004). A statistically significant difference (*p* = 0.001) in LUSBI profiles was observed between the groups of patients divided according to CVP categories. *Conclusion*: The integration of the LUSBI protocol into the differential diagnosis of dyspnea has been shown to be very effective in confirming or excluding a cardiac cause of the disease in patients.

## 1. Introduction

Regardless of the available diagnostic tools and considering its multifactorial etiology, a significant percentage of dyspneic patients whose complaints basically have a cardiac origin are initially misclassified (14–29%), which has significant implications for the further course of the disease [1]. Analyzing the contribution of various imaging methods in evaluating patients clinically presenting with dyspnea, existing research strongly supports lung ultrasound, which has a high efficiency compared to standard imaging techniques, such as chest X-ray and even thoracic computed tomography (CT) [2]. In terms of heart failure, ultrasound B lines (equivalent to B Kerley interstitial lines, which are described on native X-rays of the heart and lungs as a sign of cardiac decompensation) make the biggest contribution to confirming a diagnosis.

What sets lung ultrasound apart from other imaging methods is certainly its efficiency, simplicity and reproducibility. Additionally, it is a cost-effective and bedside method, which can be particularly advantageous in managing critically ill patients (the device is brought to the patient, rather than vice versa) [3]. The increasing use of lung ultrasound in managing patients who present clinically with varying degrees of respiratory distress has led to the need for standardizing this method in everyday practice. A decade after the First International Consensus Conference on the Application of Lung Ultrasound (LUS), new recommendations have been defined which take into account not only clinical implications but also safety, educational and technical aspects. These recommendations are the result of multidisciplinary work conducted by a large number of experts from different fields and stem from the analysis of the application and overall knowledge of LUS over the last decade [4]. The integration of lung ultrasound into differential diagnostic approaches for patients with varying degrees of respiratory distress has been achieved through several diagnostic–therapeutic protocols (BLUE, FALLS and E-FAST) [5]. Studies have shown that combining multiple diagnostic tools significantly increases the accuracy of patient classification [6]. Regarding interstitial lung syndromes, it is essential to clearly differentiate between hemodynamic and non-hemodynamic interstitial syndromes. Key sonographic features of interstitial lung syndromes are B lines, whose number, symmetry and distribution determine the nature of the underlying cause of the disease [7]. Studies have shown that there is a clear correlation between a non-B profile on lung ultrasound and pulmonary arterial wedge pressure. Additionally, very significant data regarding central venous pressure can be obtained through non-invasive sonographic assessment of the inferior vena cava (IVC) [8]. The primary aim of this study was to create a diagnostic–therapeutic protocol, LUSBI (lung ultrasound/BREST score/inferior vena cava), that integrates ultrasound characteristics of the lungs with risk stratification of acute heart failure in the form of BREST scores, as well as data on central venous pressure approximately assessed by ultrasound measurement of the IVC. This was all carried out with the goal of providing a differential diagnostic approach to dyspnea of cardiac and non-cardiac origin. The biochemical marker used to assess the effectiveness of the LUSBI protocol in determining the cardiac nature of the disease’s cause was NT pro BNP [9].

## 2. Materials 

### 2.1. Study Population

This cross-sectional study was conducted in the Emergency medicine department with the educational center of the Community health care center of Banja Luka, Bosnia and Herzegovina, in the period between September 2022 and September 2023. The study included 80 patients with chronic cardiovascular and pulmonary conditions, such as cardiomyopathy, arterial hypertension, diabetes mellitus, obesity, COPD and bronchial asthma. After meeting the inclusion criteria, the patients were divided into two groups, experimental and control groups, according to the presence or absence of dyspneic complaints at the time of enrollment. The total number of patients included in the study was 40 patients per group.

All participants signed written informed consent. Information about the study, the possibility of withdrawing at any time and confidentiality were included in a letter accompanying the questionnaire.

Demographic characteristics (such as sex, age and smoking status) were collected by self-report in the questionnaire. Clinical characteristics, as well as sonographic measurements, were collected by the researchers at the time of enrollment and included arterial blood pressure, heart rate, respiration, body temperature, hemoglobin oxygen saturation (pulse oximetry), anthropometric measurements (body weight and height, with the aim of calculating body mass index), electrocardiographic record and comorbidities.

Inclusion Criteria for the Study:

- Age over 18 years, for both sexes.
- Duration of dyspnea within 7 days prior to enrollment.
- Previously healthy or diagnosed with heart and lung diseases, except for specific exclusions cited below.

Exclusion Criteria for the Study:

- Under 18 years of age.
- Patients with tumors of the respiratory tract (lungs and bronchi).
- Patients who have experienced recent cardiac decompensation within the last 4 weeks.
- Patients who have been hospitalized due to asthma or obstructive lung disease within the last 4 weeks.
- Patients with emphysema of the lungs.
- Patients with liver cirrhosis accompanied by portal hypertension.
- Patients with neuromuscular diseases causing chronic respiratory insufficiency.
- Patients with recent chest trauma or polytrauma.

The patient evaluation process was carried out in several steps:

1. Initial procedures

After meeting the criteria for inclusion in the research, each subject underwent a clinical examination, and the following parameters were determined: arterial blood pressure, heart rate, respiration, body temperature, hemoglobin oxygen saturation (pulse oximetry), anthropometric measurements (body weight and height, with the aim of calculating body mass index) and electrocardiographic record. Laboratory blood tests were performed: complete blood count, differential blood count, C reactive protein, NT pro BNP, urea and creatinine.

2. Creation of LUSBI profiles: Each LUSBI profile is composed of three variables: sonographic lung profile (A, B, AB, C and PLAPS), central venous pressure and BREST score. In the following text, the characteristics of the individual profiles of the LUSBI protocol are described (the clinical implications of the profiles, such as the LUSBI protocol, are schematically shown in the Supplementary Materials section).

**A0 profile**: Sonographic bilateral A line with negative BREST score (0–5 points) and inferior vena cava (IVC) diameter ≤ 21 mm and/or a collapsibility index above 50% at that diameter (IVC CI above 50%).

**A1 profile**: Sonographically, bilateral A lines along both factors are positive; positive BREST score with 6 more points and IVC CI below 50% with vena cava diameter ≤ 21 mm or maximum vena cava diameter > 21 mm, regardless of IVC CI.

**A PLAPS 0 profile**: Bilateral sonographic A lines with posterolateral unilateral or bilateral consolidation and/or effusion with both factors negative, with a negative BREST score (0–5 points) and an inferior vena cava (IVC) diameter of ≤21 mm and/or a collapsibility index above 50% at that diameter (IVC CI above 50%).

**A PLAPS 1 profile:** Bilateral sonographic A lines with posterolateral unilateral or bilateral consolidation and/or effusion with both factors positive; positive BREST score (6 or more points) and IVC CI below 50% with diameter of vena cava ≤ 21 mm or max diameter of vena cava > 21 mm, regardless of IVC CI.

**AB0 profile:** A sonographic profile on one hemithorax B profile on another hemithorax, with a negative BREST score (0–5 points) or an inferior vena cava (IVC) with a diameter of ≤21 mm and/or a collapsibility index above 50% at that diameter (IVC CI above 50%).

**AB1 profile:** A sonographic profile on one hemithorax B profile on another hemithorax, with a BREST score of 6 or more points and IVC CI below 50%, with diameter of vena cava ≤ 21 mm or max diameter of vena cava > 21 mm, regardless of IVC collapsibility index.

**B0 profile:** Bilateral sonographic B lines with a negative BREST score (0–5 points) and an inferior vena cava (IVC) with a diameter ≤21 mm and/or a collapsibility index above 50% at that diameter (IVC CI above 50%).

**B1 profile:** Bilateral sonographic B lines and 1 of 2 positive factors; positive BREST score with 6 or more points or IVC CI below 50% with diameter ≤ 21 mm or max diameter IVC > 21 mm, regardless of IVC collapsibility index. 

**B2 profile:** Bilateral sonographic B lines and both positive factors; positive BREST score (6 or more points) and IVC CI below 50% with diameter of vena cava ≤ 21 mm or max diameter of vena cava > 21 mm, regardless of IVC collapsibility index.

**B PLAPS 0 profile:** Sonographic B lines bilaterally and posterolaterally (unilateral or bilateral consolidation and/or effusion), both factors listed above are negative; BREST score of 0–5 points and IVC max diameter ≤ 21 mm or IVC CI above 50% at that diameter.

**B PLAPS 1 profile**: B PLAPS profile with 1 of the 2 factors listed above positive; positive BREST score with 6 or more points or max diameter VCI > 21 mm unrelated to IVC CI or IVC CI below 50% with IVC diameter ≤ 21 mm.

**B PLAPS 2 profile:** B PLAPS profile with both factors listed above positive; BREST score of 6 or more points and IVC max diameter > 21 mm unrelated to IVC CI or IVC CI below 50% with IVC diameter ≤ 21 mm.

**C0 profile:** Unilateral or bilateral consolidation on the lungs (translobar or non-translobar) and both factors listed above negative (negative BREST score with 0–5 points) and inferior vena cava (IVC) with a diameter of ≤21 mm and/or a collapsibility index above 50% at that diameter (IVC CI above 50%).

**C1 profile:** Unilateral or bilateral lung consolidation (translobar or non-translobar), and both factors listed above positive; BREST score of 6 or more points and IVC max diameter > 21 mm unrelated to IVC CI or IVC CI below 50% with IVC diameter ≤ 21 mm.

Depending on the measured diameters of the inferior vena cava (IVC) and its collapsibility index, the central venous pressure (CVP) values are classified into three categories:

- Normal CVP (0–5 mmHg): Maximum diameter of IVC ≤ 21 mm, collapsibility index > 50%.
- Intermediate elevated CVP (5–10 mmHg):
  (a) Maximum diameter of IVC ≤ 21 mm, collapsibility index < 50%;
  (b) Maximum diameter of IVC > 21 mm, collapsibility index > 50%.

- Elevated CVP (10–20 mmHg): Maximum diameter of IVC > 21 mm, collapsibility index < 50% [8].

The collapsibility index of the inferior vena cava is calculated by the formula: VCI CI = VCI max diameter − VCI min diameter/VCI max diameter × 100%.

### 2.2. BREST Score

Risk stratification for heart failure, according to the BREST score, is based on the following:

- Anamnestic data: age, sudden dyspnea, onset of symptoms and orthopnea;
- Risk factors: previous episode of heart failure, myocardial infarction or chronic obstructive pulmonary disease;
- Clinical variables: inspiratory crackles in the lungs or “pitting” pretibial edema;
- ECG abnormalities: ST segment abnormalities or atrial fibrillation/flutter [10].

Numerical values of individual elements of the BREST score are expressed as one (1) for all factors, except for sudden dyspnea, a previous episode of heart failure and lung inspiratory crackles, which are expressed as two (2), and chronic obstructive pulmonary disease, which subtracts two points from the total score, that is, it has a numerical value of minus two (−2) points [10].

Outside of the LUSBI protocol, the BREST score selects patients into three categories based on the risk for heart failure. Subjects with ≤3 points have low risk for heart failure, those with 4–8 points have medium risk, and patients with high risk are those with ≥9 points.

Patients whose maximum numerical score (integrated into the LUSBI profile) has a value of 0–5 points are marked as negative in the context of the BREST score. Those with a maximum numerical score (integrated into the LUSBI profile) of >5 points are marked as positive in the context of the BREST score.

### 2.3. Medical Decision Making

Medical decision making is based on the existence of clearly defined profiles of the LUSBI protocol (each profile of the LUSBI protocol is described in detail, as are its clinical implications, and the procedure with the patient is shown schematically in the Supplementary Materials section).

## 3. Diagnostic Methods

### 3.1. Ultrasound of Lungs and Pleura

The examination was conducted on an ultrasound machine, the ALPINION E CUBE 7, (Alpinion medical systems, Seoul, Republic of Korea) with a corresponding linear and convex probe. Depending on the clinical condition of the patient, the examination was performed in supine position and in the semi-sitting position [4,5]. Regarding the method of ultrasound analysis, the so-called 12-points method was used, analyzing 6 points on each hemithorax, 4 points on the anterior and 2 points on the posterior thoracic wall.

### 3.2. Ultrasound of the Inferior Vena Cava

The examination was performed with a convex probe (3–5 Hz), with the patient in the supination position, and the liver was used as an acoustic window to access the inferior vena cava. Measurement of the diameters of the inferior vena cava during the respiratory cycle (maximum during expiration and minimum during inspiration) was performed at the level of the entrance to the right atrium, or about 1 cm cranial from the entrance of the hepatic veins to the inferior vena cava. The ultrasound examination was performed in B mode (a double window for both phases of the respiratory cycle) or M mode.

## 4. Statistical Analysis

Descriptive and analytical statistical methods were used. Regarding the methods of descriptive statistics, measures of central tendency and measures of variability were used, namely, arithmetic means with standard deviations and relative numbers for categorical variables. Normality of distribution was determined with the Shapiro–Wilk test. Regarding analytical statistical methods, the following methods were used to assess the significance of differences: the non-parametric alternative of the *t*-test of independent samples, the Mann–Whitney test, the non-parametric alternative of the one-factor analysis of variance (ANOVA) and the Kruskal–Wallis test, with additional Dunn–Bonferroni post hoc analysis. Among the non-parametric tests, the chi-square test was used. Spearman’s correlation analysis was used for association analysis. The usual *p*-value < 0.05 was taken as the level of statistical significance for differences, while a *p*-value < 0.010 was taken as the level for highly statistically significant differences. The SPSS version 21.0 program package (Statistical Package for Social Sciences SPSS 21.0 Inc., Armonk, NY, USA) was used for statistical data processing.

## 5. Results

This research included 80 patients, of whom 55% were female. The average age of the patients included in the study was 66.41 ± 11.01 years. The patients were divided into two age groups, a younger group from 37 to 65 years of age (41.3%) and an older group from 66 to 86 years of age (58.7%). Nearly half of the respondents were smokers (45%), and 77.4% had overweight or obesity. Besides obesity, the most common comorbidity was hypertension (80%), while arrhythmias were the least frequent (13.8%). The most significant demographic and clinical characteristics of the respondents are shown in Table 1.

**Figure 1:**
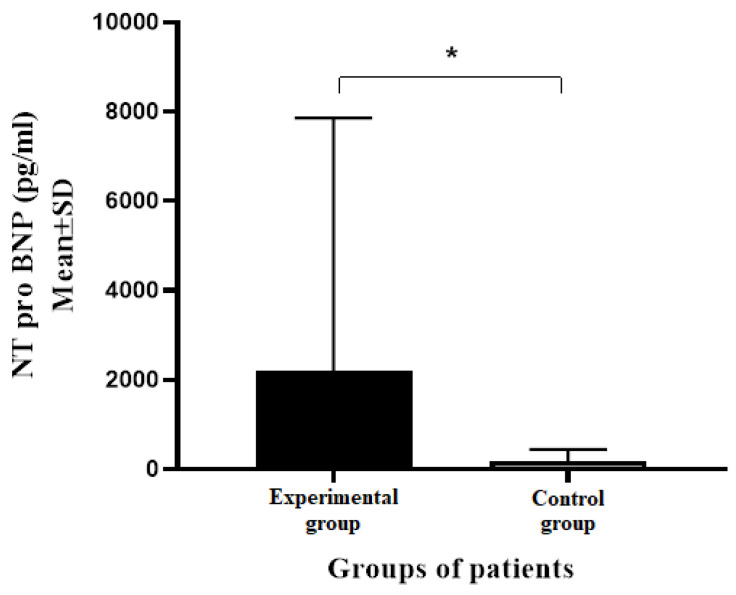
Average NT pro BNP values in experimental and control groups of patients. NT pro BNP—N-terminal pro Brain Natriuretic Peptide, AV—Average Value, SD—Standard Deviation; * *p* < 0.001 (Mann–Whitney test).

Statistically more significant differences in the average values of NT pro BNP were noted between the experimental and control groups of patients (*p* < 0.001). The patients from the experimental group had significantly higher average values of NT pro BNP compared to subjects from the control group (2184.57 ± 5672.14 pg/mL vs. 179.93 ± 264.50 pg/mL, respectively) (Figure 1).

**Figure 2:**
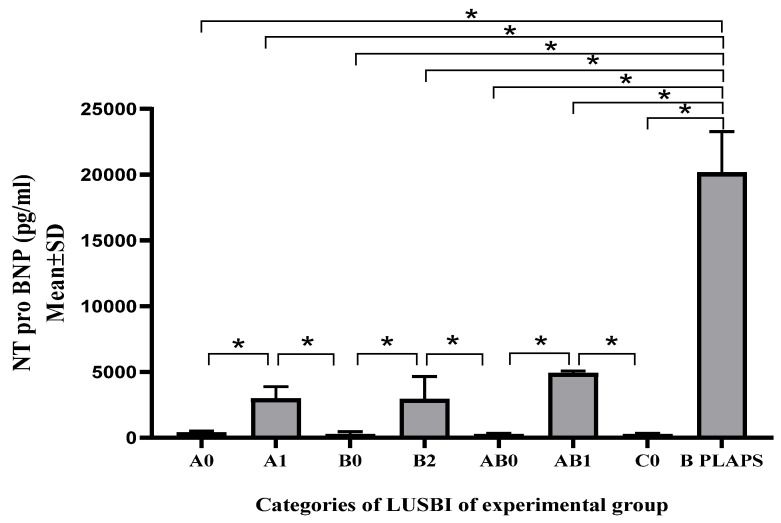
Average values of NT pro BNP in experimental group between groups of patients divided according to LUSBI protocol. AV—Average Value, SD—Standard Deviation; Kruskal–Wallis test with additional Dunn–Bonferroni post hoc analysis, * *p* < 0.001.

**Figure 3:**
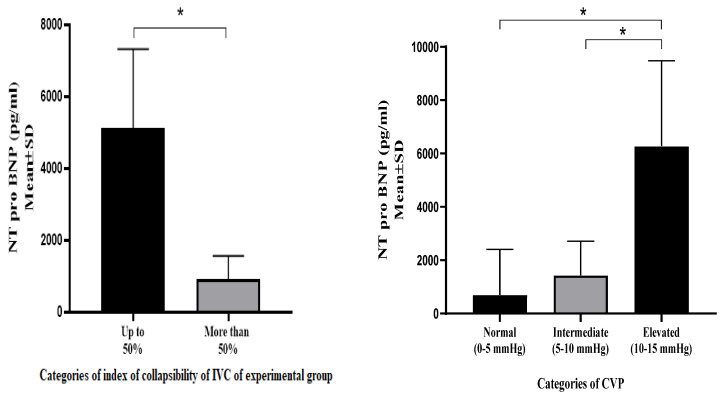
NT pro BNP distribution according to VCI CI and CVP in experimental group, * *p* < 0.001.

Within the experimental group, significant differences were observed in the NT pro BNP values according to the LUSBI profiles. No statistically significant differences were observed within the control group. The experimental group of patients who had an IVC collapsibility index less than 50% had a higher average value of NT pro BNP compared to subjects whose collapsibility index values were more than 50% (5117.00 ± 2200 pg/mL vs. 927.81 ± 631.22 pg/mL; *p* < 0.001) (Figure 2 and Figure 3).

A statistically significant difference was observed between the experimental and control groups regarding the distribution of the LUSBI profiles, categories of BREST scores and CVP measured according to the collapsibility index and the maximum diameter of the inferior vena cava (Table 2, Table 3 and Table 4). Statistically higher average values of NT pro BNP were observed in obese patients and in those with advanced-stage chronic kidney disease (CKD) (*p* < 0.001).

## 6. Discussion

In order to ensure the efficient classification of patients clinically presenting with dyspnea, particularly in terms of interstitial lung syndromes, the current research integrated lung sonography patterns with BREST scores and approximately measured central venous pressure as significant predictors of severe heart failure. This approach has proven to be entirely justified when the distribution of NT pro BNP values within the profiles of the LUSBI protocol is examined.

The LUSBI protocol is methodologically based on the analysis of 12 points (the so-called 12-points method), with 6 points on each hemithorax, 4 on the anterior and 2 on the posterior thoracic wall. The BLUE protocol, a widely used form of lung ultrasound in patients presenting clinically with an acute respiratory insufficiency, is formulated in the form of the so-called four-point method of ultrasound analysis. According to recently revised recommendations from the International Consensus Conference on Lung Ultrasound Application, it is necessary for ultrasound protocols that include lung ultrasound as an element to be fully adapted in their structure to the characteristics of the examined population. Besides the ability to evaluate the most significant lung diseases, the LUSBI protocol is absolutely adapted to different phenotypes of acute heart failure, as only in this way can complete efficiency, safety and widespread use in emergency departments be achieved for patients with the described issues.

The primary objective of incorporating the LUSBI protocol into clinical practice is to enhance the assessment of patients with various phenotypes of acute heart failure using lung ultrasound. Additionally, it aims to more effectively differentiate between hemodynamic and non-hemodynamic interstitial pulmonary syndromes. Current research about the role of lung ultrasound in patients with heart failure is exclusively related to a unique sonographic pattern—the B profile. Integration of BREST scores and CVP assessment into sonographic profiles of the lungs enables positive selection of patients with acute heart failure in the absence of B profiles, which was not possible before (only the B profile was a sonographic pattern of heart failure—pulmonary edema). Beyond the scope of high-grade pulmonary congestion, there is an impression that the efficiency of isolated lung ultrasounds in addressing other forms of acute heart failure is low (the absence of pulmonary congestion, known as “dry lungs”). The knowledge of the efficiency of the BREST score in risk stratification for acute heart failure, validated by Basset and colleagues, has also been confirmed by other studies. Outside the categories of the BREST score that sugest the absence of acute heart failure—the low-risk category (prevalence of AHF 5.7%)—and the presence of acute heart failure—the high-risk category (prevalence of AHF 79.1%)—a significant number of patients remain in a “grey zone”, necessitating the integration of the BREST score with other diagnostic tools [10]. The justification for the ultrasound assessment of the inferior vena cava as a predictor of central venous pressure is supported by recommendations from the American Society of Echocardiography [11]. Cho J. and colleagues have observed similar findings regarding the relationship between large venous vessels (the inferior vena cava, the internal jugular vein and the common femoral vein) and estimated central venous pressure [12]. Meanwhile, Parenti and colleagues indicate the lesser significance of the inferior vena cava collapsibility index relative to its maximum diameter in the evaluation of central venous pressure, which has also been confirmed by current research [13]. Considering the distribution of NT pro BNP values among the patient groups, where the previously described variables were positive in the absence of significant pulmonary congestion (non-B profiles with positive BREST scores and high CVP values), it is evident that NT pro BNP values were significantly higher compared to the groups in which the aforementioned variables were negative (A0 profile, AB0 profile and C0 profile). The stated knowledge affects the reduction in the number of negatively selected patients in emergency departments regarding the presence of acute heart failure, which greatly influences the further course of the disease. Obstructive lung syndromes (acute exacerbation of COPD, asthma and acute bronchitis), both in the symptomatic and asymptomatic phases of the disease, have an identical sonographic pattern in the lungs—A profile. In accordance with the above, the interpretation of the results of this protocol is not limited exclusively to LUSBI profile patterns, but recommendations are given for other medical procedures (laboratory analyses). This explains why the largest number of subjects selected in both groups had an A profile, which could create the impression of selection bias (especially in the control group). That is, even subjects with different phenotypes of acute heart failure (in the absence of fully developed cardiogenic pulmonary edema) did not have a different sonographic pulmonary profile. This underscores the importance of integrating additional variables with lung ultrasound profiles to promptly identify individuals at risk of acute heart failure. Besides the diagnostic significance, the application of the LUSBI protocol can achieve a certain prognostic impact (safe discharge in the case of a non-B profile where the aforementioned variables were negative) and therapeutic significance (modification of diuretic therapy based on the degree of pulmonary congestion and the level of estimated central venous pressure). This achieves complete comprehensiveness in relation to the examined clinical issue—patient selection (AHF (yes/no)), distribution (safe discharge/admission), modification of therapeutic procedures (diuretic therapy and need for ventilatory support). Yamanoglu and colleagues highlight the contribution of ultrasound assessment of the IVC to the need for hospitalization of patients with AHF, as well as the evaluation of the effects of diuretic therapy [14]. Additionally, Zanatta and colleagues observed that 42.5% of patients who clinically presented with dyspnea due to exacerbation of chronic obstructive pulmonary disease or AHF and who underwent lung ultrasound had a different therapeutic regimen compared to those whose therapy was guided by clinical characteristics (higher doses of furosemide for “wet lungs”—B profile—or metilprednisolone for “dry lungs”—A profile) [15]. It is important to highlight that this research, for the first time, integrates lung ultrasound into a unique diagnostic protocol which is not entirely based on sonographic markers of the lungs and very effectively differentiates hemodynamic from non-hemodynamic interstitial lung syndromes.

Considering the relatively small sample size included in our research, we advise caution in applying the LUSBI protocol clinically and interpreting its results until the validity of the results of the current research is verified on a larger sample. In this regard, the LUSBI protocol does not rely exclusively on the sonographic patterns of the lungs when making a medical decision but gives recommendations as to the direction in which laboratory diagnostics should go (inflammatory parameters and NT pro BNP) in order to enable a more efficient selection of subjects analyzed by this protocol. Unlike other ultrasound applications, there is no need for any patient preparation (regarding ultrasound window quality). No highly sophisticated equipment is required for this inspection, and everything can be performed with a uniform convex probe. In addition, the LUSBI protocol does not include the use of Doppler ultrasound, and its great advantage is the possibility of performing examinations at the patient’s bedside (mobile emergency medical teams).

The parameters integrated into the sonographic profiles do not require any blood analysis, nor the application of other imaging methods. Therefore, clinical examination, anamnestic data and ultrasound analysis are sufficient to create a profile and implement protocols.

## 7. Conclusions

This research was a pilot study that sought to highlight the importance of integrating variables associated with lung ultrasound (such as heart failure risk stratification and CVP assessment) in order to expand the application of lung ultrasound in daily practice, particularly for differentiating interstitial lung syndromes. In order to gain a more comprehensive understanding of the effectiveness of the LUSBI protocol and its implementation in subjects with different clinical characteristics and treatment modalities, the results of the current study need to be validated through research conducted in hospital emergency departments, specifically through a multicenter study. The greatest clinical contribution of the LUSBI protocol is expected within the entities that create the greatest diagnostic dilemmas, namely, the overlapping syndromes of heart failure and respiratory insufficiency shown in chronic obstructive pulmonary disease and pneumonia.

## Figures and Tables

**Table 1:** Demographic and clinical characteristics (n = 80).

Age, mean (±SD) range	66.41 ± 11.01
Younger group (37–65 years), n (%)	41.3
Older group (66–88 years), n (%)	58.7
Gender, n (%)	
Male	45.0
Female	55.0
Smoking, n (%)	
Yes	45.0
No	55.0
BMI categories (kg/m^2^), n (%)	
Underweight (<18.5)	1.3
Normal weight (18.6–24.9)	21.3
Overweight (25–29.9)	37.4
Obesity (>30)	40.0
Blood pressure (mmHg), mean (±SD) range	
Systolic	150.00 ± 21.25
Diastolic	82.31 ± 12.08
Mean arterial pressure	105.42 ± 13.35
Sp02 (pulse oximetry), %	95.36 ± 2.00
Complete blood count, mean (±SD) range	
Erythrocytes (3.87–5.68 × 10^12^/L)	6.26 ± 14.13
Hemoglobin (120–175 g/L)	140.15 ± 22.56
Hematocrit (35–50%)	42.46 ± 4.45
Leukocytes (3.71–10.67 × 10^9^/L)	8.05 ± 2.49
Platelets (150–450 × 10^9^/L)	229.25 ± 65.44
Biochemistry, mean (±SD) range	
Blood urea nitrogen (2.8–7.2 mmol/L)	7.38 ± 3.95
Creatinine (58–110 mmol/L)	97.30 ± 28.92
Glycemia (4.1–6.1 mmol/L)	7.29 ± 3.33
C-reactive protein (<5 mg/L)	7.30 ± 17.62
eGFR modified according to BMI (mL/min), mean (±SD) range	69.43 ± 26.24
Comorbid conditions, n (%)	
Arrhythmia	13.8
Arterial hypertension	80.0
Asthma/COPD	20.0
Cardiomyopathy	38.0
Diabetes mellitus	36.3
Obesity	77.5

SD—Standard Deviation; eGFR—estimated Glomerular Filtration Rate, BMI—Body Mass Index, COPD—Chronic Obstructive Pulmonary Disease.

**Table 2:** Distribution of patients according to categories and average values of BREST scores in experimental and control groups.

BREST Score	Experimental Group (n = 40)		Control Group (n = 40)		Complete (n = 80)		*p*
	n	%	n	%	n	%	
**BREST score categories**							
Absence of HF	14	35.0	39	97.5	53	66.3	<0.001 *
Possible HF—low risk	11	27.5	1	2.5	12	15.0	
Possible HF—high risk	13	32.5	0	0.0	13	16.3	
Presence of HF	2	5.0	0	0.0	2	2.5	
**BREST score, AV ± SD**	4.47 ± 2.60		1.00 ± 0.98		2.73 ± 2.62		<0.001 **

HF—Heart Failure, AV—Average Value, SD—Standard Deviation; * chi-square test, ** Mann–Whitney test; *p*—statistical significance, significant *p*-values bolded.

**Table 3:** Distribution of patients according to LUSBI profiles between experimental and control groups.

LUSBI Profiles	Experimental Group (n = 40)		Control Group (n = 40)		Complete (n = 80)		*p*
	n	%	n	%	n	%	
**LUSBI profiles**							
A0	16	40.0	33	82.5	49	61.3	
A1	7	17.5	0	0.0	7	8.8	
B0	1	2.5	0	0.0	1	1.3	
B2	3	7.5	0	0.0	3	3.8	0.003 *
AB0	8	20.0	7	17.5	15	18.8	
AB1	2	5.0	0	0.0	2	2.5	
C0	1	2.5	0	0.0	1	1.3	
B PLAPS 2	2	5.0	0	0.0	2	2.5	

IVC—Inferior Vena Cava; * chi-square test; *p*—statistical significance, significant *p*-values bolded.

**Table 4:** Distribution of patients according to CVP approximately measured from collapsibility index and maximum diameter of IVC between experimental and control groups.

CVP Approximately Measured from Collapsibility Index and Maximum Diameter of IVC	Experimental Group (n = 40)		Control Group (n = 40)		Complete (n = 80)		*p*
	n	%	n	%	n	%	
**CVP categories**							
Normal (0–5 mmHg)	25	62.5	34	85.0	59	73.8	
Intermediate (5–10 mmHg)	5	12.5	3	7.5	8	10.0	0.050 *
Increased (10–15 mmHg)	10	25.0	3	7.5	13	16.3	

CVP—Central Venous Pressure, IVC—Inferior Vena Cava; * chi-square test; *p*—statistical significance, significant *p* values bolded.

## Data Availability

The original contributions presented in the study are included in the article/Supplementary Materials, further inquiries can be directed to the corresponding author/s.

## Supplementary Materials

The following supporting information can be downloaded at: https://www.mdpi.com/article/10.3390/medicina60091521/s1, Figure S1: LUSBI protocol.
