# Supplementary material for: The LUSBI Protocol (Lung Ultrasound/BREST Score/Inferior Vena Cava)—Its Role in a Differential Diagnostic Approach to Dyspnea of Cardiogenic and Non-Cardiogenic Origin
Source: Medicina (Kaunas). 2024 Sep 18;60(9):1521. doi: 10.3390/medicina60091521 (PMC11433694; doi:10.3390/medicina60091521)

SUPPLEMENTARY MATERIAL

Fundamentals of ultrasound of the lungs, lungs and pleura, nomenclature of ultrasound artifacts

There are only a few clearly defined ultrasound signs (artifacts) important for the interpretation of examination results, the most significant of which is the pleural line. The common characteristic of almost all ultrasound signs on the lungs is that they are directly related to the pleural line and have a certain dynamic.

Ultrasound characteristics of bony structures (ribs), but also the air with which the lungs are filled, as well as some liquid, make it impossible to clearly visualize the lung parenchyma itself. In contrast to it, the parietal and visceral pleura can be seen more clearly, as a hyperechoic line several millimeters thick, located horizontally in the space that limits the posterior amplification (acoustic shadow) of ultrasound signals originating from the ribs. Those three structures together form the bat sign (bat sign).

Since there are several milliliters of serous fluid between the parietal and visceral pleura in normal circumstances, the visceral pleura can move freely through the parietal pleura. This dynamic sign is indicated as lung sliding on the B ultrasound mode, i.e. the beach sign on the M mode (beach sign). And the lines, as a component of a regular ultrasound finding on the lungs, represent horizontal hyperechoic lines several millimeters thick.

They are at exactly the same distance from each other, and are parallel to the pleural line. They are basically a reverberation artifact. B lines, due to their appearance, also known as comet tail, appear in the form of hyperechoic vertical lines starting from the pleural line, and have a dynamic component (following respiratory movements). They arise at the point of contact between air and water surfaces in the lungs, i.e. interlobular or lobar septa (interstitial pneumonia, initial phase of cardiogenic pulmonary edema, non-cardiogenic pulmonary edema, i.e. interstitial alveolar syndrome).

They completely "wipe" the A lines. In order to mark them as a pathological finding, it is necessary that there are 3 or more B lines in one intercostal space. As a result of the inflammatory process affecting the lung parenchyma, consolidations are formed inside the lungs, and they take on an ultrasound characteristic that corresponds to another parenchymatous organ (e.g. liver), that is, ultrasound waves are absorbed. This is registered as a hyperechoic or heteroechoic field within the

lung parenchyma, and is called a sign of hepatization (tissue like sign, hepatisation of lungs). If it is a part of the parenchyma that is consolidated, then it is usually separated from the remaining "healthy" lung parenchyma by jagged, irregular hyperechoic lines that represent the shred sign.
Considering the characteristics of the liquid, in the sense of movement within an environment towards the place of the lowest point of that environment due to the force of gravity, the accumulation of effusion within the pleural space is most often seen within the phrenicocostal sinuses. In this regard, the anechoic zone localized between the diaphragm (on the side of the liver or spleen), the lung parenchyma, and the thoracic wall is considered a pleural effusion. The dynamics of the lung towards the thoracic wall is determined by the size of the effusion, so that quantitatively small effusions will allow for almost complete expansion of the lungs during inspiration towards the thoracic wall. sinusoid sign. In B mode, an anechoic effusion limited laterally with acoustic shadows of the ribs, anteriorly by the chest wall and posteriorly by the visceral pleura is called the quad sign.
In relation to congestive heart failure, the most significant ultrasound characteristic detected in lung and pleural examinations is the presence of B-lines, distributed bilaterally along the thoracic wall (significant number being 3 or more per lung field), with or without signs of effusion in the phrenicocostal sinuses [16-17].
Description of basic ultrasound profiles of lung and pleural examination:

- A profile: bilaterally anteriorly present A-lines, with pleural sliding
- A' profile: bilaterally anteriorly present A-lines, absent pleural sliding
- B profile: bilaterally anteriorly present B-lines, with pleural sliding
- B' profile: bilaterally anteriorly present B-lines, absent pleural sliding
- A/B profile: A-lines present on one hemithorax, B-lines present on the opposite
- C profile: presence of consolidation of part or entire lobe of the lung (lung hepatization, tissue-like sign), with or without pleural sliding
- PLAPS: presence of consolidation and/or pleural effusion in the posterolateral segment of the thoracic wall

Clinical implications

- COPD/asthma: A profile, with negative PLAPS and no signs of deep vein thrombosis
- Pneumonia: A profile + PLAPS (with no signs of deep vein thrombosis); A/B, C, B' profile.

- Pulmonary embolism: A profile + clinical or sonographic signs of deep vein thrombosis of the lower extremities
- Pulmonary edema: B profile
- Pneumothorax: A' profile (further diagnostics required - lung point+, lung pulse-, M mode) [5].

References

16. Rinaldi, L.; Milione, S.; Fascione, M.C.; Pafundi, P.C.; Altruda, C.; Di Caterino, M.; Monaco, L.; Reginelli, A.; Perrotta, F.; Porta, G.; et al. Relevance of lung ultrasound in the diagnostic algorithm of respiratory diseases in a real-life setting: A multicentre prospective study. *Respirology* **2020**, *25*, 535–542. https://doi.org/10.1111/resp.13659. PMID: 31373748.
17. Gundersen, E.A.; Juhl-Olsen, P.; Bach, A.; Rostgaard-Knudsen, M.; Nielsen, B.R.R.; Skaarup, S.H.; Petersen, H.Ø.; Fjølner, J.; Poulsen, M.G.G.; Bøtker, M.T. PrehospitaL Ultrasound in Undifferentiated DyspnEa (PreLUDE): A prospective, clinical, observational study. *Scand. J. Trauma Resusc. Emerg. Med.* **2023**, *31*, 6. https://doi.org/10.1186/s13049-023-01070-4. PMID: 36740691; PMCID: PMC9899351.

LUSBI PROTOCOL

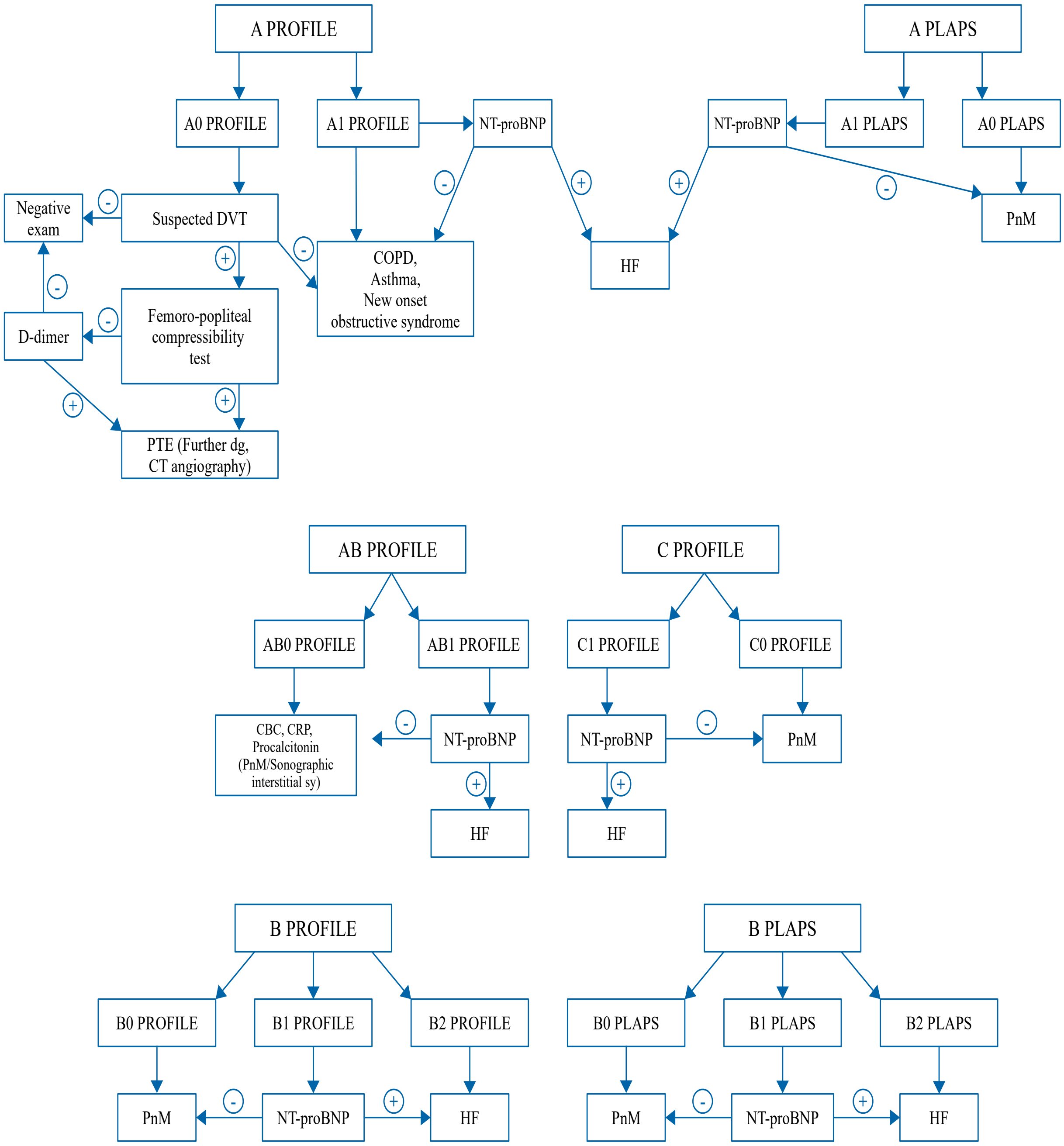

Supplement: Supplementary file 1 [file medicina-60-01521-s001.zip › medicina-3163341-supplementary.pdf]
